# Targeting Mitochondrial Function in *Plasmodium falciparum*: Insight into Antimalarial Drugs and the Emerging Role of *Saccharomyces cerevisiae* as a Model System

**DOI:** 10.3390/ijms26189150

**Published:** 2025-09-19

**Authors:** Sara Greco, Graziana Assalve, Paola Lunetti, Kassoum Kayentao, Antoine Dara, Dario Scaramuzzi, Vincenzo Zara, Alessandra Ferramosca

**Affiliations:** 1 Department of Experimental Medicine, University of Salento, I-73100 Lecce, Italy; sara.greco@unisalento.it (S.G.); graziana.assalve@unisalento.it (G.A.); paola.lunetti@unisalento.it (P.L.); vincenzo.zara@unisalento.it (V.Z.); 2 Parasites and Microbes Research and Training Center, University of Science, Techniques and Technologies, Point G, Bamako P.O. Box 1805, Mali; kayentao@icermali.org (K.K.); tonydara@icermali.org (A.D.); 3 R-Evolution Worldwide Srl Impresa Sociale, I-73024 Maglie, Italy; d.scaramuzzi@revolutionworldwide.community

**Keywords:** malaria, mitochondria, *Plasmodium falciparum*, *Saccharomyces cerevisiae*, antimalarials

## Abstract

Malaria remains a major global health threat, particularly in low- and middle-income countries, where children under five and pregnant women are most vulnerable. Despite notable progress in reducing malaria-related morbidity and mortality, the rise of drug-resistant *Plasmodium falciparum* strains continues to undermine eradication efforts. In this context, the parasite’s mitochondrion has emerged as a promising target for novel antimalarial therapies due to its essential role in parasite viability throughout all life cycle stages and its marked structural and biochemical differences from the human counterpart. This review highlights recent advances in the development of compounds targeting mitochondrial function in *P. falciparum* and discusses the utility of *Saccharomyces cerevisiae* as a powerful model organism for antimalarial drug discovery. Owing to its shared eukaryotic features, genetic tractability, and capacity for heterologous expression of parasite mitochondrial proteins, *S. cerevisiae* offers a cost-effective and experimentally accessible platform for elucidating drug mechanisms and accelerating therapeutic development.

## 1. Introduction

According to the latest World Health Organization (WHO) report, an estimated 263 million malaria cases occurred globally in 2023, across 83 endemic countries. This disease was responsible for approximately 627,000 deaths, with the majority of cases occurring among pregnant women and children under five years of age in low- and middle-income countries [1].

Malaria is caused by protozoan parasites of the genus *Plasmodium*, with *Plasmodium falciparum* being the most prevalent and virulent species [2]. Although artemisinin-based combination therapies (ACTs) remain the frontline treatment recommended by the WHO, the emergence and spread of artemisinin (ART)-resistant *P. falciparum* strains highlight the urgent need for novel therapeutic targets, drugs, and treatment strategies.

The mitochondrion of *P. falciparum* has attracted increasing scientific interest as an antimalarial target due to its central role in parasite viability and development and its significant divergence from the host organelle, enabling selective therapeutic intervention. Functionally, this organelle supports a wide range of essential cellular processes in the parasite, including ATP production, regulation of redox homeostasis, and the biosynthesis of pyrimidines and heme [3].

Despite stage-specific fluctuations in its metabolic activity throughout the parasite life cycle in the human host, from the liver stages, through the intraerythrocytic asexual stages, to the sexual (gametocyte) stages, the mitochondrion remains an indispensable organelle across all of these phases. Consequently, the increasing focus on this organelle perfectly aligns with the recent paradigm shift in antimalarial drug discovery, which prioritizes the development of multi-stage, mechanism-driven therapies [4].

While current antimalarial drugs primarily act on the asexual blood stages responsible for clinical disease manifestations, there is a pressing demand to identify next-generation antimalarials with broader activity profiles that are able not only to treat the active infection but also to prevent and block the transmission, supporting long-term malaria eradication efforts [5].

In this evolving research landscape, *Saccharomyces cerevisiae* has emerged as a robust eukaryotic model for mitochondria-targeted antimalarial studies. With its genetic tractability, metabolic versatility, and conservation of core mitochondrial pathways yeast provides an ideal platform for in-depth functional studies and high-throughput screening (HTS) of compounds that disrupt essential mitochondrial processes in *P. falciparum*. The yeast system not only facilitates the identification of specific mitochondrial molecular targets but also allows for the assessment of stage-selective drug activity by simulating the distinct metabolic states associated with different phases of the *P. falciparum* life cycle in the human host. Indeed, by modulating growth conditions, such as oxygen levels and carbon-source utilization, yeast metabolism shifts between glycolytic and respiratory states, similarly to what occurs in the parasite’s various life stages.

This review provides a comprehensive overview of the most promising newly discovered or developed mitochondrial-targeting antimalarial compounds, focusing on their molecular mechanisms and stage-specific effects. Additionally, the role of *S. cerevisiae* is explored as a versatile tool for both drug discovery and mechanistic characterization in the context of mitochondrial inhibition.

## 2. Role of Mitochondria in the Parasite Life Cycle

### 2.1. General Overview of the Parasite Life Cycle

*P. falciparum* has a complex life cycle involving both humans and *Anopheles* mosquitoes. Transmission begins when a mosquito injects sporozoites into the human bloodstream. These migrate to the liver, infect hepatocytes, and multiply during the pre-erythrocytic stage (liver stage), producing thousands of merozoites.

**Figure 1:**
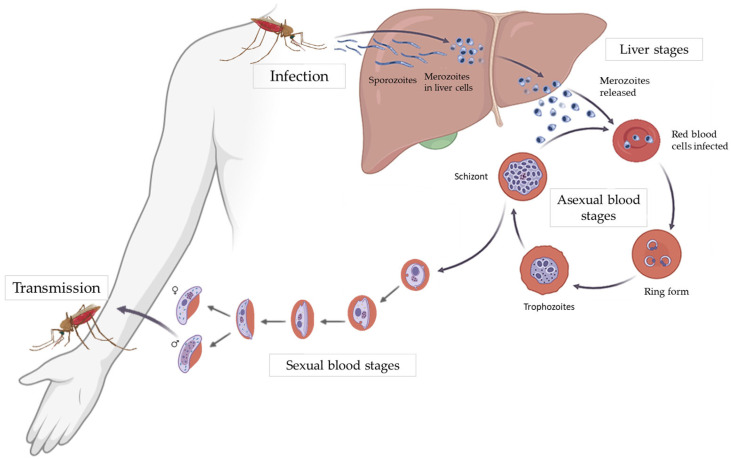
*Plasmodium falciparum* life cycle. Adapted from [7], licensed under CC BY 4.0.

Merozoites then enter the bloodstream and invade red blood cells (RBCs), starting the erythrocytic stage, where they replicate, cause cell lysis, and release new merozoites. Some differentiate into gametocytes, which are taken up by another mosquito during a blood meal. Inside the mosquito, they develop into sporozoites that reach the salivary glands, ready to infect a new host [6] (Figure 1).

### 2.2. Mitochondrial Metabolism of the Parasite in the Liver Stage

Studying *P. falciparum* during its liver stage is particularly difficult, as this phase is asymptomatic and inaccessible in natural human infections. Consequently, much of our understanding comes from rodent malaria models (*Plasmodium yoelii* and *Plasmodium berghei*), engrafted mice, and limited in vitro systems using human hepatocytes [8]. After entering hepatocytes, sporozoites mature over 6–7 days, undergoing extensive genome and organelle replication without cytokinesis, leading to a multinucleated schizont. Cytokinesis occurs at the end of this stage, producing thousands of merozoites that enter the bloodstream.

During liver-stage development, the parasite’s mitochondrion increases in size and complexity, displaying tubular cristae characteristic of active, metabolically adaptive organelles [9,10]. However, the metabolic role of mitochondria in this stage remains only partially understood.

To investigate this, Tarun et al. performed transcriptomic and proteomic analyses on *P. yoelii* liver stages. Their study revealed increased expression of genes involved in the tricarboxylic acid (TCA) cycle and mitochondrial electron transport chain (mETC), suggesting enhanced mitochondrial activity to meet biosynthetic and energetic demands [11].

Further insights came from integrating genetic screens of barcoded *P. berghei* mutants with transcriptomic data, identifying nine TCA-cycle-related genes as essential for liver-stage development such as the ones encoding for aconitase and α-ketoglutarate dehydrogenase [12,13]. Glucose likely feeds into glycolysis and the TCA cycle, driving ATP production and biosynthesis [14].

Collectively, these findings suggest that during liver-stage growth, *Plasmodium* adopts a hybrid metabolic strategy—scavenging nutrients such as glucose and amino acids from host hepatocytes, while synthesizing other molecules like fatty acids to support its rapid proliferation.

### 2.3. Mitochondrial Metabolism of the Parasite in the Asexual Blood Stages

During the 48 h asexual intraerythrocytic cycle of *P. falciparum* (comprising ring, trophozoite, schizont, and merozoite phases) the parasite’s single mitochondrion undergoes morphological changes, enlarging and branching, yet it remains acristate in all stages [15,16].

The absence of cristae in the mitochondrion suggests a limited capacity for oxidative phosphorylation, implying that the parasite relies predominantly on host-derived glucose and glycolytic pathways for energy production, a metabolic adaptation that has been documented in multiple studies [17,18].

Although the mitochondrion plays a limited role in ATP production during the asexual blood stages of *P. falciparum*, it remains essential for parasite survival. There are conflicting views regarding the operation of the TCA cycle during the asexual blood stages. Some studies suggest that it functions in a branched manner, with intermediates such as 2-oxoglutarate being diverted mainly for biosynthetic purposes rather than for complete oxidative metabolism. In contrast, other evidence points to a canonical, cyclical function of the pathway. This controversy stems from differences in experimental design, isotope-labeling interpretations, and the parasite’s context-dependent metabolic flexibility.

**Figure 2:**
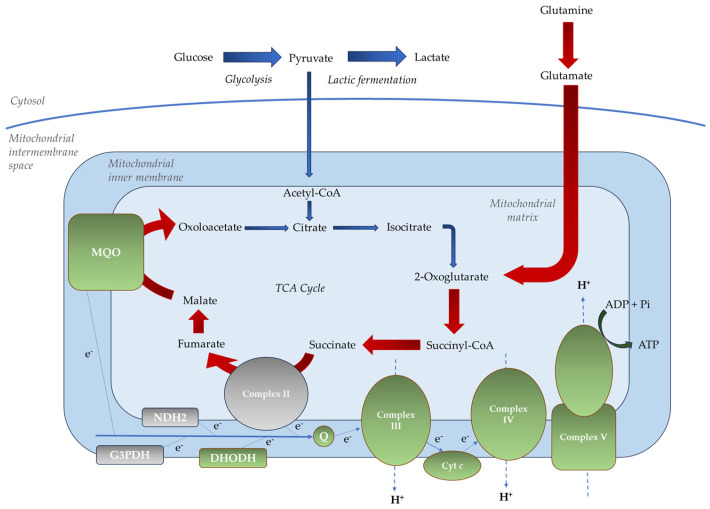
The main carbon and mitochondrial metabolic pathways in *P. falciparum* during the asexual blood stages. In these stages, most glucose is metabolized through glycolysis to produce lactate, with only a small proportion of pyruvate being converted to acetyl-CoA to fuel the tricarboxylic acid cycle (TCA) cycle. Indeed, glucose generates a minor carbon flux from oxaloacetate to isocitrate. Conversely, glutamate produces a major flux from 2-oxoglutarate to malate. Blue arrows indicate glucose metabolism, while red arrows represent glutamine metabolism. Thicker arrows indicate the major carbon flux, while thinner ones represent the minor flux. Essential components of the mitochondrial electron transport chain (mETC) are shown in green, and dispensable ones in gray. Cyt *c*, cytochrome *c*; DHODH, dihydroorotate dehydrogenase; G3PDH, glycerol 3-phosphate dehydrogenase; MQO, malate-quinone oxidoreductase; NDH2, type II NADH dehydrogenase; Pi, inorganic phosphate.

Early metabolomic studies proposed that the TCA cycle in the parasite might not operate as a conventional, unidirectional pathway. Instead, it appeared to function in a branched manner, diverging at the intermediate 2-oxoglutarate, as follows: a reductive branch proceeding in reverse from 2-oxoglutarate through isocitrate, citrate, and oxaloacetate to malate, and an oxidative branch proceeding from 2-oxoglutarate through succinyl-CoA, succinate, and fumarate to malate [19]. However, more recent studies have shown that *P. falciparum* expresses all of the enzymes required for a complete, canonical TCA cycle during its intraerythrocytic development [17]. This cycle is sustained by carbon skeletons derived from host glucose and glutamine. Specifically, a minor flux from oxaloacetate to isocitrate is maintained through the conversion of pyruvate to acetyl-CoA via the branched-chain ketoacid dehydrogenase complex (*Pf*BCKDH). In contrast, a major flux from 2-oxoglutarate to malate is driven by carbon sourced from glutamine [17,20,21] (Figure 2).

Overall, the parasite demonstrates remarkable metabolic plasticity, regulating nutrient fluxes (glucose, amino acids, and lipids) and rerouting central carbon metabolism to adapt to fluctuating host microenvironments. This flexibility enables *P. falciparum* to balance energy generation with biosynthetic demands, ensuring survival and replication in response to variations in host cell resources, oxygen availability, and immune pressures.

Genetic knockout studies indicate that most TCA cycle enzymes are not essential for *P. falciparum* survival during the asexual blood stages, reinforcing the idea that the cycle has a limited role in energy production [22]. Instead, the TCA cycle likely serves biosynthetic and redox functions, contributing to mETC activity, de novo pyrimidine synthesis, purine salvage, and heme biosynthesis by supplying key intermediates such as 2-oxoglutarate, succinyl-CoA, and oxaloacetate [23].

The *P. falciparum* mETC includes the following:

- Non-proton motive quinone reductases, such as dihydroorotate dehydrogenase (*Pf*DHODH), malate-quinone oxidoreductase (*Pf*MQO), glycerol 3-phosphate dehydrogenase (*Pf*G3PDH), type II NADH dehydrogenase (*Pf*NDH2, alternative complex I), and succinate dehydrogenase (*Pf*SDH, complex II);
- Proton motive respiratory complexes, including ubiquinol-cytochrome *c* oxidoreductase (complex III), cytochrome *c* oxidase (complex IV), and ATP synthase (complex V) [24] (Figure 2).

Although all these components are expressed during the asexual blood stages, not all are essential. *Pfndh2* gene deletion via CRISPR/Cas9 did not affect intraerythrocytic development [25], consistent with findings in *P. berghei* [26]. Similarly, knockout studies targeting *pfsdh* revealed that its absence did not impair blood-stage parasite growth [27,28].

In contrast, complexes III, IV, and V are essential. Complex III is critical for reoxidizing ubiquinol to support the activity of *Pf*DHODH, which is indispensable for de novo pyrimidine biosynthesis, a pathway essential to the parasite, as it cannot salvage pyrimidines [21,22]. Complexes III and IV also maintain the mitochondrial membrane potential (Δψ_m_) necessary for metabolite transport and mitochondrial function [29]. Although Complex V is not required for ATP production during the asexual blood stages [30], genetic disruption of its subunits has proven unsuccessful, suggesting it plays a vital role independent of oxidative phosphorylation [31].

### 2.4. Mitochondrial Metabolism of the Parasite in the Sexual Blood Stages

During each asexual replication cycle, a small fraction (<10%) of *P. falciparum* ring-stage parasites commit to sexual development, a process called gametocytogenesis [32]. Gametocytes mature through five stages (I–V) over 10–12 days. Mature stage V gametocytes are taken up by mosquitoes, where they differentiate into male (microgametes) and female (macrogametes) gametes in the mosquito midgut [32,33,34].

Gametocyte development involves a programmed remodeling of central carbon metabolism. Unlike asexual blood stages, which rely mainly on anaerobic glycolysis, gametocytes shift toward oxidative metabolism, increasing mitochondrial dependence [17].

This metabolic shift reflects the changing energy demands and environmental conditions faced by gametocytes. While asexual stages rapidly generate ATP through glycolysis in the glucose-rich bloodstream, gametocytes require more efficient and sustained energy production to support prolonged maturation and survival within both the human host and mosquito vector. Hence, the switch to mitochondrial oxidative phosphorylation provides a higher ATP yield and supports biosynthetic pathways crucial for gametocyte development and transmission [17].

These changes are supported by upregulation of the TCA cycle and oxidative phosphorylation genes [17,21], as well as by mitochondrial enlargement into complex multi-lobed structures with tubular cristae [16,35]. This structural adaptation facilitates increased mETC activity and aligns mitochondrial function with the metabolic demands of gametocytes, making mitochondrial morphological changes both a consequence and a facilitator of the metabolic reprogramming.

**Figure 3:**
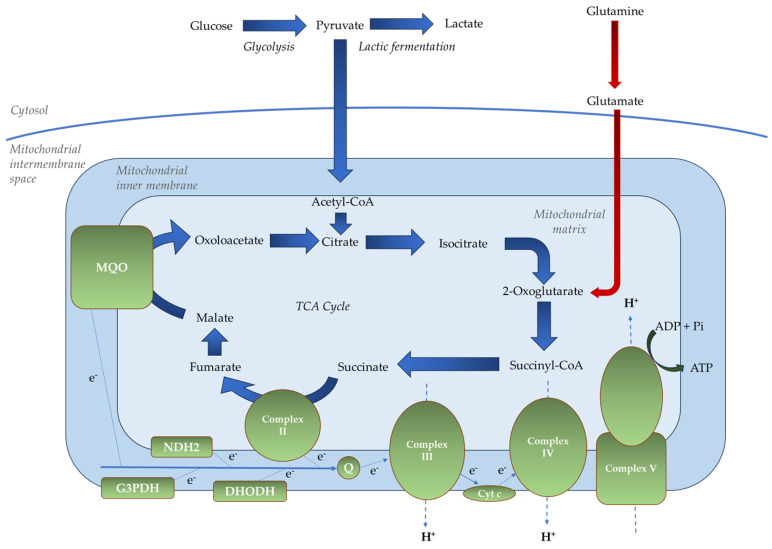
The main carbon and mitochondrial metabolic pathways in *P. falciparum* during the sexual blood stages. In these stages, a switch to a predominant glycolytic flux into a highly active TCA metabolism occurs, accompanied by a reduced carbon flux from glutamate. Blue arrows indicate glucose metabolism, while red arrows represent glutamine metabolism. Thicker arrows indicate the major carbon flux, while thinner ones represent the minor flux. Essential components of the mETC are shown in green.

Metabolomic analyses show gametocytes actively run the TCA cycle using glucose (primary) and glutamine (secondary) as carbon sources, opposite to the asexual stage pattern [17,28] (Figure 3).

Disruption of TCA cycle genes impairs gametocyte development or mosquito-stage transmission [17,21,36]. Indeed, deletion of *P. falciparum* aconitase (*pfaco*) results in parasites that arrest at stage III, failing to produce mature gametocytes. Similarly, deletion of α-ketoglutarate dehydrogenase (p*fkdh*) yields morphologically normal gametocytes that cannot form oocysts when ingested by mosquitoes. Deletion of isocitrate dehydrogenase (*pfidh*) blocks development beyond stages III/IV [28,36]. Moreover, while several mETC components are dispensable in asexual stages, they are essential in gametocytes. Disruption of genes such as *pfndh2* and *pfsdh* leads to defective gametocyte development.

## 3. Antimalarial Agents That Target Mitochondrial Function

Atovaquone is the first and only clinically approved antimalarial specifically targeting the *Plasmodium* mitochondrion. As a ubiquinone analog, it binds to the cytochrome *b* subunit of complex III, disrupting the mETC and compromising parasite energy metabolism. Chemically classified as a hydroxynaphthoquinone, atovaquone specifically targets the quinol oxidation (Q_o_) site of complex III [37].

When administered as a fixed-dose combination with proguanil (commercially known as Malarone), atovaquone is widely used for both the treatment of uncomplicated malaria in adults and children and for chemoprophylaxis in travelers. Its excellent safety profile makes it suitable for vulnerable populations, including pregnant women and children, and for mass drug administration campaigns [38]. However, rapid emergence of atovaquone-resistant parasites threatens its long-term efficacy, underscoring the urgent need to develop and clinically validate new mitochondrial-targeting antimalarials.

While resistance to atovaquone arises from mutations in cytochrome *b*, additional mechanisms of resistance have been reported for other mitochondrial targets. For example, resistance to *Pf*DHODH inhibitors, such as DSM265, can result from point mutations in the *pfdhodh* gene that reduce drug-binding affinity, or from gene amplification events that increase *Pf*DHODH expression [39]. Moreover, metabolic compensation, such as the upregulation of alternative metabolic pathways or enhanced utilization of host-derived metabolites, can support parasite survival despite inhibition of mitochondrial function, as observed in some ART-resistant parasites [40]. Understanding these adaptive mechanisms is critical for the development of next-generation antimalarials with durable efficacy.

### 3.1. Agents Targeting the mETC

**Figure 4:**
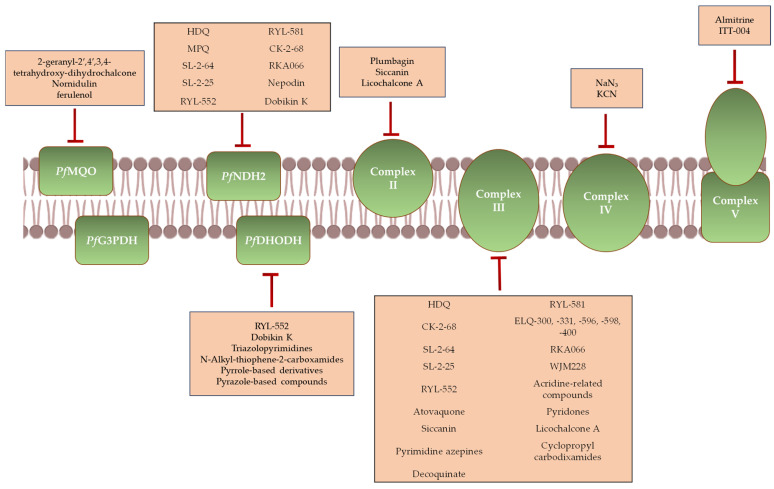
Antimalarial compounds acting on distinct components of the mETC. HDQ, MPQ, CK-2-68, SL-2-64, SL-2-25, RYL-552, RYL-581, RKA066, WJM228, ELQ-300, ELQ-331, ELQ-596, ELQ-598, and ELQ-400 are endochin-like quinolones. Atovaquone, Nepodin, and Plumbagin are naphthoquinones. Siccanin and Dobikin K are terpene compounds.

The majority of antimalarial compounds target mETC complexes and dehydrogenases (Figure 4).

#### 3.1.1. Inhibitors of PfNDH2

*Pf*NDH2 is a single-subunit mitochondrial enzyme that functions as an alternative to the multi-subunit complex I in humans. It catalyzes the transfer of electrons from NADH to ubiquinone, thus exhibiting NADH—ubiquinone oxidoreductase activity. Its structural divergence from the human enzyme makes it an attractive target for antimalarial drug development [41].

Several endochin-like quinolones (ELQs) have shown potent inhibitory activity against *Pf*NDH2, with all compounds evaluated in vitro and some also assessed in vivo. Among them, HDQ and its derivatives (CK-2-68, SL-2-64, and SL-2-25) are particularly promising. Initially considered to be *Pf*NDH2-specific, these compounds were later found to also inhibit complex III, indicating a dual-target mechanism [25,42,43].

HDQ exhibits moderate-to-strong in vitro activity (IC_50_ = 70 nM for *Pf*NDH2; 19 nM for complex III) but is limited by high lipophilicity (CLogP = 6.5) [42,44]. Structural modifications aimed at improving pharmacokinetics led to derivatives such as CK-2-68, which maintains potent *Pf*NDH2 inhibition with reduced activity against complex III, though its lipophilicity remains similar. SL-2-25 offers balanced inhibition of both enzymes and a lower lipophilicity (CLogP = 4.2), while SL-2-64 shows exceptional potency against *Pf*NDH2 (IC_50_ = 4 nM) and favorable physicochemical properties [38,42].

Another notable class is the RYL series, characterized by a multitarget profile. RYL-552 was first described as a non-competitive *Pf*NDH2 inhibitor (IC_50_ = 3.73 nM) and later shown to also bind the Q_o_ and quinone reduction (Q_i_) sites of complex III, with potential activity against *Pf*DHODH [41,45,46]. In murine models, RYL-552 effectively cleared parasitemia at various doses (10–90 mg/kg). Its optimized analog, RYL-581, exhibits markedly improved potency (EC_50_ = 0.056 nM) and in vivo efficacy, outperforming atovaquone and leading to complete parasite clearance in infected mice [41]. RKA066 is another dual-site inhibitor, active on both *Pf*NDH2 and the Q_i_ site of complex III [47].

In contrast to these multitarget inhibitors, a yeast-based screen identified MPQ as a selective *Pf*NDH2 inhibitor, highlighting its potential for higher target specificity [43].

Natural products have also been explored. Nepodin, a naphthol compound, selectively inhibits *Pf*NDH2 in vitro and significantly suppresses parasitemia in vivo [48]. More recently, Dobikin K, a sesquiterpenoid, was found to inhibit *Pf*NDH2 in a concentration-dependent manner and disrupt the Δψ_m_ in *P. falciparum* [49].

#### 3.1.2. Inhibitors of *Pf*SDH

*Pf*SDH exhibits notable structural differences compared to its mammalian counterpart. While the mammalian enzyme comprises four subunits (Fp, Ip, CybL, and CybS) only the flavoprotein (Fp) and iron–sulfur cluster protein (Ip) subunits have been identified in *P. falciparum*, with no apparent homologs for the membrane anchor subunits [50,51]. This divergence underscores *Pf*SDH as a potential target for selective antimalarial therapy.

The antibiotic Siccanin has emerged as a potent in vitro *Pf*SDH inhibitor, showing nanomolar activity against the enzyme and low micromolar inhibition of complex III. Notably, Siccanin exhibits no detectable effect on mammalian complexes at high concentrations, supporting its selectivity for the parasite [51].

Other small molecules have also demonstrated *Pf*SDH inhibitory activity. Plumbagin, a hydroxynaphthoquinone, selectively targets *Pf*SDH and shows promising in vitro antimalarial effects, although its in vivo efficacy remains limited [52,53].

Licochalcone A, a natural chalcone, inhibits both *Pf*SDH and complex III in vitro, with a stronger effect on complex III [54,55].

#### 3.1.3. Inhibitors of Complex III

Complex III is a well-established antimalarial target. Inhibitors of this complex typically act at the Q_o_ or Q_i_ sites of the cytochrome *b* subunit by mimicking ubiquinone/ubiquinol. Their selectivity for *P. falciparum* arises from species-specific differences in the coenzyme Q (CoQ-binding site residues [56]. These compounds span several chemical classes, from ELQs to cyclopropyl carboxamides.

ELQs primarily target complex III, though many also inhibit other components of the mETC. Among Q_i_ site inhibitors, ELQ-300 is a standout candidate, with nanomolar potency (IC_50_ = 0.56 nM), high selectivity (>18,000-fold over human complex III), and favorable safety and metabolic profiles [57,58,59,60]. Due to poor solubility and low oral bioavailability, the prodrug ELQ-331 was developed, demonstrating improved pharmacokinetics and progressing toward once-weekly prophylactic use [59]. ELQ-596, a biphenyl analog, improves potency against resistant *P. falciparum* strains and its prodrug, ELQ-598, was optimized for better ADME (absorption, distribution, metabolism, and excretion) properties [61]. Targeting the Q_o_ site, compounds such as WJM228 have shown potent activity [58,62,63]. ELQ-400 binds both Q_o_ and Q_i_ sites, exhibiting strong dual-site inhibition [64,65].

Acridine-related compounds also demonstrate significant activity against complex III. WR 249685 is a potent Q_o_ site inhibitor (IC_50_ = 3 nM) with higher selectivity than atovaquone [57]. T111, the most potent acridone reported, is thought to act on both Q_o_ and Q_i_ sites [66,67].

Pyridones are validated scaffolds for complex III inhibition. GW844520 and its successor GSK932121 inhibit the Q_i_ site, showing high potency and preclinical efficacy. However, their clinical development was halted due to cardiac and off-target toxicity in mammals, respectively [42,47,57,68,69].

Another promising class includes pyrimidine azepines. PyAz90 has demonstrated strong in vitro and ex vivo activity as a novel Q_o_ site inhibitor, with partial overlap with atovaquone’s binding region but distinct molecular interactions [70].

Cyclopropyl carboxamides were identified through a Seahorse XFe96 metabolic screening of the MMV (Medicines for Malaria Venture) Pathogen Box. Among these, MMV024397 emerged as a potent Q_o_-site inhibitor, with reduced efficacy observed in atovaquone-resistant *P. falciparum* strains, supporting its proposed mechanism of action [56]. Further screening identified several structurally related analogs, including WJM280, W466, and W499, all active in the low nanomolar to submicromolar range without detectable cytotoxicity [71].

The quinolone decoquinate is another significant inhibitor of complex III, likely targeting the Q_o_ site, with strong efficacy in vitro and in vivo, as well as limited cross-resistance with atovaquone [42,72].

#### 3.1.4. Inhibitors of Complex IV

Complex IV has recently emerged as a promising but underexplored antimalarial target. In *P. falciparum*, it is susceptible to classical inhibitors like sodium azide (NaN_3_) and potassium cyanide (KCN). However, their lack of selectivity between parasite and host enzymes leads to inhibition of human mitochondrial respiration, significantly limiting their therapeutic potential [73,74]. This highlights the need for parasite-selective inhibitors with improved safety profiles.

#### 3.1.5. Inhibitors of Complex V

Few inhibitors of complex V have been identified to date. Among them, almitrine, originally developed as a respiratory stimulant, has shown moderate in vitro antimalarial activity, with IC_50_ values of 13.2 ± 5.6 µM (chloroquine-resistant FCM 29) and 12.6 ± 3.6 µM (chloroquine-sensitive L-3) [55,75].

Another example is ITT-004, a derivative of Basic Blue 3. While the parent compound is highly potent but cytotoxic, ITT-004 maintains antimalarial efficacy with significantly reduced cytotoxicity in vitro and in vivo [76].

#### 3.1.6. Inhibitors of *Pf*DHODH

*Pf*DHODH links the mETC to de novo pyrimidine biosynthesis by catalyzing dihydroorotate to orotate, the pathway’s rate-limiting step. Given pyrimidines’ essential role in nucleic acid, phospholipid, and glycoprotein synthesis, *Pf*DHODH is a validated and druggable target.

DSM265, a triazolopyrimidine and the first *Pf*DHODH inhibitor in clinical development, shows potent in vitro activity (IC_50_ = 0.024 µM), excellent selectivity (>4000-fold vs. human DHODH; IC_50_ > 100 µM), and favorable pharmacokinetics in preclinical models [77,78,79]. Phase Ia/Ib trials support its potential for single-dose treatment and weekly chemoprophylaxis, and it has advanced to Phase IIa studies as both monotherapy and in combination therapy in adults [80]. DSM421, a second-generation compound of the same chemical class, has slightly lower potency (IC_50_ = 0.053 µM) but superior pharmacokinetic (PK) properties, including better solubility, plasma exposure, and reduced clearance [77].

Using HTS and medicinal chemistry, Booker et al. identified N-alkyl-5-(1H-350 benzimidazol-1-yl)-thiophene-2-carboxamides as selective *Pf*DHODH inhibitors [81,82]. Among these, Genz-667348 demonstrated potent inhibition (IC_50_ = 0.022 µM) but was halted at the late lead stage due to off-target toxicity [42,79]. However, its analog Genz-669178, with similar potency (IC_50_ = 0.04 µM), showed improved safety and PK, and was effective in multiple murine malaria models [42].

Palmer et al. reported two pyrrole-based *Pf*DHODH inhibitors, DSM705 and DSM873, with IC_50_ values of 0.095 µM and 0.069 µM, respectively. Both showed strong in vivo efficacy and favorable PK in rodents and SCID mice, supporting continued development [83,84].

Building on pyrrole scaffold, pyrazole-based derivatives were explored, leading to DSM1465—a highly potent and selective inhibitor (IC_50_ = 0.015 µM) that outperformed DSM265 by 7-fold in vitro. DSM1465 also showed robust efficacy in SCID mouse models and has a predicted human half-life of 115 h, suggesting the potential for single-dose chemoprevention lasting up to 30 days [85].

#### 3.1.7. Inhibitors of *Pf*MQO

MQO’s absence in humans makes it a unique antimalarial target.

Nornidulin, a fungal metabolite, inhibited *Pf*MQO (IC_50_ = 51 µM) and suppressed *P. falciparum* proliferation (IC_50_ = 44.6 µM) without mammalian cytotoxicity [86].

A screen of 113 known mETC inhibitors identified ferulenol as the most potent (IC_50_ = 57 nM), acting as an uncompetitive inhibitor forming a dead-end complex with *Pf*MQO [87].

Hidayati et al. isolated 2-geranyl-2′,4′,3,4-tetrahydroxy-dihydrochalcone from *Artocarpus altilis*, which showed potent antimalarial activity (IC_50_ = 0.17 µM), low cytotoxicity (CC_50_ = 19.7 µM), and a high selectivity index (115.8) [88].

### 3.2. Other Mitochondrial Targets

Beyond *Pf*MQO and *Pf*SDH, other TCA enzymes are attractive drug targets.

*Pf*ACO is selectively inhibited by sodium fluoroacetate (NaFAc), causing citrate accumulation and depletion of downstream TCA metabolites in *P. falciparum* [17,89].

*Pf*FH, a class I fumarate hydratase, is potently inhibited by mercaptosuccinic acid (Ki in the nanomolar range). The human enzyme, a class II hydratase, is structurally unrelated, making *Pf*FH a highly selective target [90].

Other mitochondria-targeting antimalarials act through indirect mechanisms. ART, for instance, is thought to disrupt mitochondrial function by causing membrane depolarization without directly targeting classical mETC components [91]. The *Plasmodium* heme biosynthesis pathway also plays a role: excess 5-aminolevulinic acid (ALA) leads to reactive oxygen species (ROS) production and parasite growth inhibition. When combined with sodium ferrous citrate (SFC), ALA shows synergistic antimalarial activity, curing 50% of *P*.-*yoelii*-infected mice and providing long-term protection, highlighting its potential for combination therapies [92,93].

## 4. Potential Therapeutic Applications of Mitochondria-Targeting Agents Across Different Stages of the Parasite Life Cycle

Historically, malaria drug development has concentrated on the asexual blood stage of *Plasmodium* parasites, the clinically relevant phase responsible for symptoms such as fever, anemia, cerebral malaria, and metabolic acidosis. These complications result from the rupture of infected erythrocytes, triggering inflammation and the release of parasitic by-products. This stage has remained the primary target for antimalarial therapies due to its direct involvement in disease pathology and accessibility to systemic drugs. Consequently, most approved antimalarials act on intraerythrocytic parasites to reduce parasite load and alleviate symptoms. However, the emergence of *P. falciparum* strains resistant to frontline treatments, including ACTs, underscores the limitations of this single-stage focus. Resistance has rapidly developed to all blood-stage antimalarials, threatening treatment efficacy and global control efforts. To address these challenges and support eradication goals, research is shifting toward developing next-generation drugs with novel mechanisms and multi-stage activity aimed not only at treating acute infection but also at preventing infection and blocking transmission.

To support this approach, the global malaria research community, introduced a framework of target candidate profiles (TCPs) and target product profiles [94]. These TCPs classify compounds based on activity against specific *Plasmodium* life stages. For *P. falciparum*, the following three are especially relevant: TCP-1—targets asexual blood stages (symptomatic); TCP-4—targets liver stages (asymptomatic); and TCP-5—targets sexual stages responsible for transmission.

Each stage represents a potential bottleneck in the parasite life cycle and, thus, a valuable target for intervention. Importantly, all human-infecting stages of *Plasmodium* depend to varying extents on mitochondrial functions essential for survival, including energy metabolism, pyrimidine and heme biosynthesis, and redox balance. The mitochondrion, therefore, presents a promising target for the development of multi-stage antimalarial agents.

Table 1 summarizes the mitochondrial targets discussed in Section 3, along with the corresponding life stages affected in the human host. This helps clarify the relationship between each molecular target and its stage-specific antiplasmodial activity.

Among mETC components, complex III is a well-established antimalarial target, essential across all three developmental stages of *Plasmodium* within the human host.

In the asexual blood stage, complex III is crucial for ubiquinone recycling, supporting *Pf*DHODH-mediated de novo pyrimidine biosynthesis, a process vital for parasite survival [28,30]. In liver and sexual stages, where mitochondrial respiration appears coupled to ATP production, complex III supports electron transport as part of core mETC function.

All known complex III inhibitors consistently demonstrate strong activity against asexual blood stages, highlighting the complex’s essentiality. Several compounds also inhibit liver and sexual stages, indicating broader utility, though in the absence of data, potential multi-stage activity cannot be ruled out.

Atovaquone, the only approved mitochondrial-targeting antimalarial, is effective at all intra-host stages: it treats symptomatic blood-stage infection, prevents liver-stage development, and blocks transmission by targeting gametocytes. It shows activity across gametocyte maturation, with IC_50_ values ranging from 24 µM for stages II–III to 5.31–50.4 µM for stages IV–V, indicating reduced potency in later stages [7].

This aligns with the general trend of greater drug susceptibility in early gametocytes (stages I–III).

Several antimalarial compounds targeting complex III have shown efficacy across the three key stages of *P. falciparum* development in humans. Among them, ELQ-300, ELQ-331, and ELQ-400 have demonstrated potent activity against liver, asexual blood, and transmission stages [59,64,65,96]. In murine models, ELQ-400 prevented both transmission and liver-stage infection at single oral doses as low as 0.08–0.1 mg/kg, and effectively cleared blood-stage parasites at 1 mg/kg [98].

The pyrimidine azepine compound PyAz90 has also exhibited multi-stage potential. Although it showed only moderate activity in vitro against *P. falciparum* gametocytes (EC_50_ > 10 μM), it significantly reduced exflagellation center formation by up to 80% at 10 μM, suggesting a transmission-blocking effect [70,101].

Similarly, compounds from the cyclopropyl carboxamide class have demonstrated broad activity across liver, blood, and sexual stages. Decoquinate, identified through a large-scale screen of 1037 compounds, emerged as a highly potent complex III inhibitor against liver stages, with an IC_50_ of 2.6 nM. It has also shown efficacy against asexual and sexual blood stages in both in vitro and in vivo settings [72].

Beyond complex III, other mitochondrial targets such as *Pf*NDH2 and *Pf*SDH are required for transmission and may play roles in liver-stage development, but they appear non-essential in asexual blood stages [26,27,35,108].

Accordingly, MPQ, a specific *Pf*NDH2 inhibitor, is ineffective against asexual parasites, and its activity in other stages remains uncharacterized. Nepodin, another *Pf*NDH2 inhibitor, has been associated with reduced parasitemia in vivo [48], though this effect may stem from off-target mechanisms. Similarly, plumbagin, a *Pf*SDH inhibitor, shows limited efficacy against asexual stages, and data for liver or gametocyte stages are lacking.

Complex IV, along with complex III, contributes to maintaining the Δψ_m_, suggesting it may be essential across all parasite stages. However, known inhibitors such as NaN_3_ and KCN are broad-spectrum complex IV inhibitors with poor specificity, and their effects have only been observed in asexual forms [73]. Experimental evidence for their activity against liver or sexual stages is currently absent.

The function of complex V varies with the parasite stage. In liver stages, glucose import suggests possible ATP production via oxidative phosphorylation, although direct experimental support is lacking [14]. In contrast, during the asexual blood stage, mitochondrial ATP generation is minimal [30], yet complex V appears essential, possibly due to alternative functions, as gene disruption attempts in *P. falciparum* have failed [31]. This is supported by observations on almitrine, which exhibits moderate in vitro activity against asexual blood stages, and ITT-004, which reduces parasitemia both in vitro and in vivo. However, no data are currently available on their efficacy in liver or sexual stages. In gametocytes, where energy metabolism supports the extensive remodeling needed for maturation and transmission, the role of complex V remains unexplored.

*Pf*DHODH, a key enzyme in pyrimidine biosynthesis, is essential in liver and asexual blood stages, but not in gametocytes [30]. DSM265, a selective *Pf*DHODH inhibitor, shows potent activity against liver stages and slows asexual-stage parasite growth, but does not clear mature gametocytes [102]. Other *Pf*DHODH inhibitors (Table 1) also demonstrate efficacy primarily against asexual stages, with occasional activity in liver stages; none show confirmed activity in sexual stages.

The TCA cycle appears largely dispensable during the asexual blood stage, with six out of eight enzymes being non-essential. The essentiality of *Pf*MQO and *Pf*FH remains debated; although early studies suggested they could not be knocked out [28], more recent CRISPR-based evidence indicates they may be dispensable [22]. In contrast, the TCA cycle is more critical in gametocyte development. Disruption of *Pf*ACO arrests gametocyte maturation at stage III and its inhibitor NaFAc has no effect on asexual stages but significantly impairs sexual-stage development [17,28,36]. Specific inhibitors of *Pf*MQO (such as 2-geranyl-2′,4′,3,4-tetrahydroxy-dihydrochalcone, nornidulin, and ferulenol) and *Pf*FH (e.g., mercaptosuccinic acid) show activity against asexual stages, though their effects in liver or sexual stages remain untested.

## 5. The Yeast *S. cerevisiae* as a Model for Malaria Research: From General Considerations to Mitochondrial Pharmacological Targeting

### 5.1. General Advantages of S. cerevisiae as a Model for P. falciparum Research

This section outlines the rationale for using *S. cerevisiae* as a robust and versatile model organism in malaria research, particularly for studying *P. falciparum* molecular biology and identifying parasite-specific genetic and metabolic pathways.

As unicellular eukaryotes, *S. cerevisiae* and *P. falciparum* share key cellular features, including a membrane-bound nucleus, Golgi apparatus, endoplasmic reticulum, and mitochondria. Despite evolutionary divergence, their mitochondria retain similar structural and functional characteristics (double membranes, internal compartmentalization, and an electron transport chain), supporting roles in ATP production, metabolism, oxidative stress response and biosynthesis of essential metabolites. These similarities are discussed in greater detail in Section 5.2.

One major advantage of yeast is its rapid, cost-effective growth in simple media. With a generation time of approximately 90–120 min, *S. cerevisiae* thrives in media containing basic nutrients [109]. In contrast, *P. falciparum* requires complex, stage-specific culture conditions, including RPMI 1640 supplemented with glucose, hypoxanthine, and reduced glutathione [110]. The parasite also depends exclusively on glucose, is unable to metabolize alternative sugars, and is highly sensitive to vitamin antimetabolites [111]. Additionally, its in vitro maintenance necessitates a low-oxygen (microaerophilic) environment to replicate erythrocytic conditions.

Yeast’s genetic tractability is another key asset. Its well-characterized genome, short life cycle, and ability to exist in haploid form allow for efficient gene manipulation, mutagenesis, and knockout studies [112]. These properties are invaluable for dissecting gene function and enable heterologous expression of *P. falciparum* proteins, particularly mitochondrial proteins with yeast orthologs, which is essential for investigating mechanisms of drug action and resistance.

Moreover, *S. cerevisiae* displays remarkable metabolic plasticity. As a facultative anaerobe, it can switch between fermentation and mitochondrial respiration depending on oxygen availability and carbon source. Under fermentable conditions like glucose, it favors fermentation in low oxygen; when grown on non-fermentable carbon sources (e.g., ethanol or acetate), it relies solely on oxidative metabolism [113,114]. This flexibility mirrors the metabolic transitions in *P. falciparum*, which shifts from glycolysis in its asexual blood stages to oxidative metabolism during liver-stage development and gametocytogenesis.

This metabolic adaptability is also relevant for understanding drug resistance. For instance, ART resistance in *P. falciparum* involves not only mutations in the *pfkelch13* gene but also significant metabolic reprogramming. Resistant parasites show increased glycolytic activity and altered TCA cycle flux, enhancing glutathione production to mitigate oxidative stress. The mitochondrial citrate/oxoglutarate carrier (*Pf*COCP) supports this by exporting key metabolites for cytosolic glutathione synthesis and NADPH generation. Simultaneously, downregulation of mETC components indicates a shift toward alternative energy pathways [40].

Finally, *S. cerevisiae* is a powerful tool for antimalarial drug discovery, particularly in the development of HTS platforms. Genetically engineered yeast strains enable the rapid testing of large chemical libraries, significantly accelerating the identification of candidate compounds [115,116]. For example, Frame et al. engineered yeast to express the *P. falciparum* purine transporter *Pf*ENT1, identifying nine inhibitors that blocked [^3^H]-adenosine uptake and parasite growth [117]. Similarly, Sweeney et al. constructed a yeast strain dependent on the parasite’s phosphate transporter *Pf*PiT, leading to the identification of two active compounds from a 21-compound library [118].

### 5.2. Functional and Structural Analogies and Divergences Between the Mitochondrion of P. falciparum and S. cerevisiae

Despite species-specific differences, the mitochondria of *S. cerevisiae* and *P. falciparum* share significant structural and functional similarities, making yeast a useful heterologous system for studying mitochondrial proteins from the parasite. These similarities enable functional complementation studies to investigate potential drug targets and stage-specific roles of mitochondrial proteins in *P. falciparum*.

A key point of comparison is the mETC. Both organisms lack the canonical multi-subunit complex I, instead relying solely on type II NADH dehydrogenases. In yeast, three such enzymes exist, Ndi1, Nde1, and Nde2, with Ndi1 and Nde1 being functionally dominant and homologous to the parasite single NADH dehydrogenase, *Pf*NDH2 [43]. Notably, Ndi1 and *Pf*NDH2 are both oriented toward the mitochondrial matrix and oxidize NADH from the TCA cycle, whereas Nde1 and Nde2 face the intermembrane space and process cytosolic NADH [119].

Complex II in both species includes conserved catalytic subunits (Sdh1 and Sdh2 in *Saccharomyces*, and *Pf*SDHA and *Pf*SDHB in *Plasmodium*), responsible for transferring electrons from succinate to ubiquinone. However, *P. falciparum* exhibits a divergent *Pf*SDHC subunit (Sdh3 in yeast) and lacks a direct homolog of Sdh4. Instead, it incorporates four unique accessory proteins (*Pf*C2AP1-4), which are absent in yeast and likely contribute to complex stability [16]. The parasite’s complex II also supports bidirectional activity, catalyzing both succinate oxidation and reverse fumarate reduction, reflecting its metabolic flexibility [53].

Complex III in *P. falciparum* (~730 kDa) is slightly larger than that of yeast (~670 kDa) and comprises 12 subunits compared to yeast’s 10. Despite this, it retains approximately 40% sequence homology [16,29,120]. The three catalytic subunits (cytochrome *b*, cytochrome *c*_1_, and the Rieske iron–sulfur protein) are conserved. Importantly, the Q_o_ site of cytochrome *b*, a known drug-binding region, is highly conserved. While yeast-specific subunits like Core 1, Core 2, and Qcr10 are absent in *P. falciparum*, the parasite contains orthologs of Qcr6–9 and unique proteins such as *Pf*MPPα/β and apicomplexan-specific *Pf*C3AP1–3 [16]. 

Complex IV in *P. falciparum* is notably larger (~570 kDa) than in yeast (~200 kDa), due to its expanded subunit composition—22 subunits versus yeast’s 12–13 [121,122]. While both species share core catalytic subunits (Cox1, Cox2, and Cox3) along with several non-catalytic components, *P. falciparum* possesses lineage-specific subunits, including *Pf*COX13, *Pf*COX14, *Pf*COX16, *Pf*COX18, *Pf*COX19, *Pf*COX24, *Pf*COX30, and five unique proteins (*Pf*C4AP1–5) absent in yeast [16].

Complex V is also largely conserved in its catalytic architecture. Both species share core F_1_ subunits (ATPα, β, γ, δ, and ε) and structural components of the peripheral stalk, such as OSCP and subunits b and d. The proton-translocating F_o_ domain also shows conservation, including subunit a, the c-ring, ATP8, ATPi/j, and ATPk. However, *P. falciparum* expresses several unique accessory subunits (*Pf*ATPTG2, G3–G4, G6, G9–G13, G15, and G17) not found in yeast, while yeast-specific subunits like ATPe, ATPf, and ATPg are absent in the parasite [16,123,124].

A notable divergence lies in the DHODH enzymes. Yeast expresses a cytosolic, fumarate-dependent type 1A Dhodh that does not require ubiquinone, while *P. falciparum* utilizes a mitochondrial, ubiquinone-dependent *Pf*DHODH. This difference precludes functional complementation of the parasite enzyme by the yeast counterpart [125].

However, recent advances in genetic engineering could provide potential strategies to overcome this limitation. For instance, heterologous expression of *Pf*DHODH in yeast strains lacking endogenous Dhodh could be optimized by fusing a yeast-specific mitochondrial targeting sequence to the parasite enzyme, to facilitate the correct mitochondrial localization of *Pf*DHODH within yeast cells. Additionally, the design of chimeric proteins combining domains from yeast and *P. falciparum* DHODHs could enhance structural compatibility and enzymatic functionality in the yeast cellular environment. Such chimeras might retain the parasite-specific catalytic site while adopting yeast-specific regions that promote proper folding, stability, and interactions with other mitochondrial proteins. These approaches have been successfully applied in other cross-species complementation studies and represent promising avenues for functional expression and drug screening applications in yeast-based models [126,127].

TCA cycle enzymes also illustrate key divergences. For example, while yeast mitochondrial malate dehydrogenase (Mdh1) and *Pf*MQO both catalyze the conversion of malate to oxaloacetate, they differ in location and cofactor used. Mdh1 operates in the matrix using NAD^+^ as an electron acceptor, whereas *Pf*MQO is membrane-bound and uses quinone, reducing it to quinol [128]. Similarly, FH differs in class and catalytic mechanism. *S. cerevisiae* expresses a class II Fh, oxygen-stable and lacking Fe–S clusters, while *P. falciparum* encodes a class I *Pf*FH that is oxygen-sensitive and contains [4Fe–4S] clusters critical for radical-based catalysis. These differences prevent functional complementation between the two enzymes [90].

Several yeast mitochondrial carriers also show potential orthology with parasite proteins. For example, Yhm2, involved in oxidative stress response in yeast, has a likely counterpart in *P. falciparum* (PlasmoDB ID PF3D7_1223800), possibly playing a similar protective role against oxidative damage [40,129,130].

### 5.3. S. cerevisiae as a Model Organism to Study the Function of P. falciparum Mitochondrial Proteins

Understanding the roles of individual *P. falciparum* proteins is crucial for uncovering metabolic pathways essential to parasite survival and for identifying new antimalarial targets. *S. cerevisiae* is a valuable model for the functional characterization of *P. falciparum* mitochondrial proteins due to its genetic manipulability and capacity for mitochondrial transformation. This system supports detailed analysis of protein localization, regulatory interactions, and function within a conserved eukaryotic mitochondrial environment.

Many *P. falciparum* mitochondrial proteins share sequence or structural homology with yeast counterparts, enabling comparative functional genomics. Functional complementation assays, expressing parasite genes in yeast strains lacking the corresponding endogenous proteins, can reveal conserved biochemical mechanisms and highlight potential therapeutic targets. This approach has been applied in several studies. Salcedo-Sora et al. characterized *Pf*CHA, a putative mitochondrial Ca^2+^/H^+^ antiporter involved in calcium homeostasis. Expression of *Pf*CHA in a Δ*vcx1* yeast mutant restored calcium sequestration following external calcium challenge, monitored through an apoaequorin-based bioluminescent assay for cytosolic Ca^2+^, Mg^2+^, and Mn^2+^ fluxes [131]. Although *Pf*CHA localizes to the mitochondria in *P. falciparum*, in yeast it targeted the vacuolar membrane but retained functionality, demonstrating the flexibility of the yeast system for studying membrane transport proteins despite differences in subcellular targeting.

Jenkins et al. examined *Pf*COQ10, the *P. falciparum* orthologue of the yeast ubiquinone-binding protein Coq10, essential for mitochondrial respiration. Despite sharing only ~27% sequence identity, *Pf*COQ10 preserved key residues for ubiquinone binding. Its expression in a Δ*coq10* yeast strain rescued growth on glycerol, a non-fermentable carbon source. Western blotting confirmed mitochondrial localization, while a truncated version lacking the mitochondrial leader sequence failed to complement the defect, highlighting the importance of correct mitochondrial import [132].

Chellappan et al. investigated *Pf*PHB1 and *Pf*PHB2, two *P. falciparum* prohibitins, highly conserved inner mitochondrial membrane proteins involved in mitochondrial DNA organization, respiratory complex assembly, and overall mitochondrial integrity. Structural modeling with Protein homology/analogY recognition engine (Phyre2) revealed strong similarity to homologs in other species, including conserved interaction motifs. Yeast two-hybrid assays confirmed interaction between *Pf*PHB1 and *Pf*PHB2, suggesting that similar prohibitin complexes may form in the parasite mitochondrion [133].

### 5.4. S. cerevisiae as a Model Organism for the Identification and Characterization of Molecular Targets of Promising Antimalarial Compounds

Given the functional and structural similarities in mitochondrial proteins between yeast and the malaria parasite highlighted in the Section 5.2, along with conserved mitochondrial pathways—such as oxidative phosphorylation, Δψ_m_ maintenance, protein import, and redox homeostasis—enable *S. cerevisiae* to make a meaningful impact on accelerating antimalarial drug discovery. Specifically, *S. cerevisiae* is a powerful tool for identifying molecular targets of antimalarial compounds, particularly through site-directed mutagenesis to pinpoint amino acid residues critical for compound binding. This approach, combined with computational docking and molecular modeling, provides insights into binding conformations and affinities, aiding drug design and optimization.

Song et al. used yeast to investigate the target of ELQ-400, leveraging the conserved Q_o_ and Q_i_ sites of cytochrome *b* between yeast and apicomplexans [64]. Yeast strains carrying *Plasmodium*-like mutations in these sites were engineered to assess ELQ-400 sensitivity. The compound strongly inhibited the yeast complex III (IC_50_ ≈ 88 nM), with mutations in either site significantly altering sensitivity. *Plasmodium*-like substitutions in the Q_i_ site increased sensitivity up to seven-fold, while Q_o_ site mutations enhanced it up to 17-fold. ELQ-400 demonstrated a distinct binding mode compared to other Q_i_ inhibitors, as evidenced by the absence of cross-resistance in Q_i_ mutants. Docking analyses showed that ELQ-400 mimics ubiquinol binding at the Q_i_ site, with key residues like M221 contributing to stabilization. At the Q_o_ site, binding is supported by a hydrogen bond with Rieske iron–sulfur protein-H181 and π–π interactions with Y279; the Y279S mutation moderately reduced sensitivity [64]. These findings confirm ELQ-400 targets both Q_o_ and Q_i_ sites, suggesting a dual inhibition mechanism effective against resistant *Plasmodium* strains.

Since the atomic structure of complex III remains unresolved, the yeast complex III crystal structure (PDB ID: 3CX5) is commonly used as a reference. Based on this, Calit et al. generated a 3D model of complex III based on the yeast structure to investigate the interaction of PyAz90 with the atovaquone binding site [70]. Docking revealed π–π interactions between PyAz90 and complex III residues F123 and F264, located near conserved residues Y268 and V259 within the atovaquone binding pocket [134]. These results indicate that PyAz90 and atovaquone bind overlapping regions of complex III, but engage distinct residues [70].

### 5.5. S. cerevisiae as a Model Organism to Characterize the Mechanism of Action and/or Resistance of Antimalarial Compounds

#### 5.5.1. Plasmodione

As part of the PlasmoPrim project, Mounkoro et al. investigated the mitochondrial and redox-related effects of Plasmodione (PD) using *S. cerevisiae* as a model [135]. The AD1–9 yeast strain, lacking several membrane transporters and thus more sensitive to xenobiotics, revealed that PD selectively inhibited respiratory growth at low micromolar concentrations, without affecting fermentative growth up to 80 μM. Despite this growth inhibition, mitochondrial respiration remained intact, as shown by normal oxygen consumption and uncoupling responses [135].

Further analysis indicated that oxidative stress plays a central role in PD toxicity. Yeast mutants deficient in oxidative defense genes such as *CTT1*, *SOD1*, and *SOD2* were more sensitive, particularly the Δ*sod2* strain. PD also inactivated yeast aconitase, a mitochondrial Fe–S enzyme susceptible to ROS, while leaving fumarase activity unaffected, indicating selective oxidative damage. Biochemical assays with isolated yeast mitochondria showed that PD and its metabolites (PD-bzol and PDO) act as electron acceptors for mitochondrial NADH dehydrogenases, with PD-bzol and PDO generating more ROS, supporting the involvement of redox cycling in PD toxicity. Unlike atovaquone, PD and most of its metabolites had little effect on the complex III [135].

Subsequent work identified mitochondrial flavoenzymes as key players in PD bioactivation. Mutations in *SDH1*, encoding the Fp subunit of the yeast succinate dehydrogenase (Sdh), conferred resistance by disrupting the FAD-binding site and reducing enzyme Sdh activity, likely impairing production of toxic metabolites. Mutations in *LPD1* and *LIP2*, affecting pyruvate and α-ketoglutarate dehydrogenase complexes, also conferred resistance, emphasizing the role of NADH-dependent redox cycling in PD activation. Biochemical evidence confirmed mitochondrial NADH dehydrogenases as major drivers of redox cycling, with Sdh catalyzing early FAD-dependent reactions [136]. These findings are relevant to *Plasmodium*, which expresses a *Pf*SDH complex. The *Pf*SDHA subunit, homologous to yeast Sdh1, is essential during late gametocyte development—stages where PD is particularly active. Transcript data show increased *pfsdha* and *pfndh2* expression in schizonts and gametocytes, consistent with stage-specific PD sensitivity. However, the presence of alternative flavoenzymes or residual *Pf*SDH activity may enable partial PD activation in early stages [136].

Overall, yeast studies highlight the central role of mitochondrial flavoproteins in mediating PD’s redox cycling and antimalarial activity.

#### 5.5.2. Proguanil

Within the same project, *S. cerevisiae* was used to investigate the mechanism of proguanil, a biguanide antimalarial used in combination with atovaquone in Malarone. Despite its long-standing clinical use, the mode of action of proguanil remains unclear. It does not inhibit the mETC directly, and its activity was hypothesized to involve disruption of the parasite’s alternative Δψ_m_ maintenance mechanism, involving the adenine nucleotide translocator (ANT), complex V, and phosphate carriers [30].

Since atovaquone inhibits complex III [35], the synergy with proguanil was thought to result from blocking both mETC-dependent and -independent Δψ_m_ pathways. Using AD1–9 yeast grown on non-fermentable media, Mounkoro et al. confirmed that proguanil acts synergistically with complex III inhibitors, including atovaquone, azoxystrobin, and ELQ-271, while showing antagonism with Plasmodione [137]. This synergy was lost in the atovaquone-resistant cytochrome *b* Y279C mutant, confirming that complex III inhibition is necessary for proguanil potentiation. Alone, proguanil had no effect on respiration, nor did it enhance atovaquone-induced inhibition [137].

Unlike complex III inhibitors, proguanil was cytotoxic rather than cytostatic. Rho^0^ mutants, lacking mitochondrial DNA and respiratory complexes, showed slightly reduced sensitivity, suggesting that neither the mETC nor Δψ_m_ maintenance is its primary target. Resistant mutants carried mutations near *PUF3*, a regulator of mitochondrial mRNA and CoQ biosynthesis. Deleting *COQ5* reduced proguanil sensitivity, while supplementation with decylubiquinone increased it. These findings indicate that CoQ levels and mitochondrial membrane properties affect drug accumulation and toxicity. The synergy with atovaquone may arise from increased mitochondrial accumulation of proguanil rather than direct Δψ_m_ disruption [137].

#### 5.5.3. ARTs

Despite widespread clinical use, the mechanisms of action of ART and its derivatives (ARTs) remain incompletely understood. Studies using *S. cerevisiae* have uncovered multiple, context-dependent effects of ART and its active derivative dihydroartemisinin (DHA) [138].

Both compounds inhibit yeast growth on non-fermentable media at low concentrations, but only DHA inhibits growth on fermentable media at moderate concentrations.

One mechanism shared by both drugs is a mitochondria-targeted, heme- and Sod1-independent effect, observed at 2–8 μM during respiratory growth. In isolated mitochondria, ARTs caused rapid, reversible depolarization of the mitochondrial membrane, independent of ROS. This effect likely involves activation by electrons from the mETC that reduce the endoperoxide bridge of ARTs, generating free radicals [138]. Supporting this, deletion of the NADH dehydrogenases genes *NDI1* and *NDE1* confers resistance, while overexpression increases sensitivity [139].

A second mechanism involves heme-mediated toxicity. Heme catalyzes ARTs activation, producing ROS that cause broad oxidative damage. While both ART and DHA react with heme, DHA is more potent, likely due to greater chemical reactivity. DHA thus retains activity under both respiratory and fermentative conditions, while ART efficacy is limited to the former [138].

A third mechanism, observed only at high ART concentrations (200–400 μM), involves Sod1-suppressible, ROS-dependent mitochondrial toxicity. Δ*sod1* mutants are highly sensitive under fermentative conditions, showing increased ROS and mitochondrial depolarization. This effect is abolished under hypoxia or in respiratory-deficient mutants and is mitigated by antioxidants or metal ions. DHA exhibits this activity too, though it is usually masked by its stronger heme-mediated effects [138].

Laleve et al. further explored ARTs effects using in vitro, in organello, and in cellulo models [140]. ARTs were shown to restore respiratory growth in a yeast strain with defective complex III assembly (*BCS1* mutation), likely by interfering with cytochrome *c*_1_ maturation and restoring a balanced complex assembly. Fluorescently labeled DHA bound reduced cytochrome *c*, suggesting activation requires reduced iron and highlighting *c*-type cytochromes as potential mitochondrial targets [140].

#### 5.5.4. Primaquine

Primaquine (PQ), one of the few antimalarials effective against all three *Plasmodium* life cycle stages, has an incompletely defined mode of action. Using *S. cerevisiae* mutants, Laleve et al. found that the Δ*sod2* strain was highly sensitive to PQ, and that antioxidant treatment or overexpression of mitochondrial oxidative stress genes *AIM32* and *MCR1* reduced this sensitivity. Overexpression of *SOD1* had no effect, confirming the importance of mitochondrial, rather than cytosolic, antioxidant defense [141].

Fe–S cluster-containing proteins were identified as PQ targets. PQ treatment selectively inactivated yeast aconitase under respiratory conditions, when ROS levels are elevated. Rho^0^ mutants were resistant, implicating mitochondrial ROS as key mediators of PQ cytotoxicity. These findings suggest that PQ exploits endogenous ROS to damage mitochondrial targets and inhibit cell growth [141].

Given that *Pf*ACO is essential in *P. falciparum* gametocytes and that Fe–S enzymes are vulnerable to oxidative inactivation, PQ likely exerts its antimalarial activity by targeting redox-sensitive mitochondrial proteins in metabolically active parasite stages.

## 6. Conclusions and Future Perspectives

Over the past two decades, remarkable progress has been made in reducing the number of malaria cases and deaths worldwide, largely driven by the introduction of innovative approaches and strategies for malaria control and elimination. Yet, despite these intensive, large-scale efforts, malaria remains a major global public health challenge, imposing a significant social and economic burden on endemic countries and undermining their health system resilience and sustainable development [1].

Adding complexity to this scenario is the emergence of *Plasmodium* strains resistant to all currently available frontline antimalarial drugs, including ACTs. This highlights the urgent need to identify new pharmacologically relevant molecular targets, develop a robust pipeline of promising candidates, and introduce novel treatments. Ideally, next-generation antimalarial drugs should not only prevent clinical disease onset and relieve symptoms but also block transmission to others, a crucial step toward the ultimate goal of malaria eradication.

This review offers a comprehensive overview of the most promising mitochondrial-targeting antimalarial compounds identified in recent years, underscoring the mitochondrion as a particularly attractive target for novel agents with potential multi-stage activity. Although mitochondrial metabolism varies across the different stages of *P. falciparum* development (hepatic, intraerythrocytic, and gametocytic), it remains essential throughout, enabling the discovery of compounds that function as chemoprophylaxis, treatment, and transmission-blocking agents.

The future outlook in this field is highly encouraging: integrating functional genomics, HTS, and advanced bioinformatics could accelerate the identification of new targets and deepen our understanding of still poorly characterized resistance mechanisms.

In this context, yeast has proven to be an extremely valuable experimental model for identifying and characterizing molecular targets, dissecting drug mechanisms of action, and predicting potential resistance pathways—even for drugs already in clinical use [135,137,138,139,141].

Yeast’s genetic versatility, combined with the conservation of many mitochondrial metabolic pathways with *Plasmodium*, enables the design of targeted and informative experiments, thereby facilitating early-stage discovery and optimization of new mitochondria-targeting antimalarial agents. Furthermore, developing genetically engineered yeast models that more accurately replicate parasite mitochondrial metabolism could enhance the translational relevance of experimental findings.

Ultimately, a deeper understanding of mitochondrial involvement in drug response may pave the way for more effective combination therapies capable of overcoming emerging drug resistance and making a tangible contribution to malaria elimination efforts.

## Figures and Tables

**Table 1:** Specific molecular targets of the mitochondrial inhibitors discussed in Section 3 and the related *Plasmodium* life stages affected in the human host. ABSs, asexual blood stages; ALA/SFC, 5-aminolevulinic acid/sodium ferrous citrate; ART, artemisinin; LSs, liver stages; ND, not detected; *Pf*ACO, *Plasmodium falciparum* aconitase; *Pf*DHODH, *Plasmodium falciparum* dihydroorotate dehydrogenase; *Pf*FH, *Plasmodium falciparum* fumarate hydratase; *Pf*MQO, *Plasmodium falciparum* malate-quinone oxidoreductase; *Pf*NADH2, *Plasmodium falciparum* type II NADH dehydrogenase; ROS, reactive oxygen species; SBS, sexual blood stages; (+), active; (−), inactive.

Compounds	Mitochondrial Target	*Plasmodium* Life Cycle Stage Target			References
		LS	ABS	SBS	
**Endochin-like quinolones**					
Hydroxy-2-dodecyl-4-(1H)-quinolone (HDQ)	*Pf*NADH2 complex III (Qi site)	ND	(+)	ND	[25,42,43,44]
7-Chloro-3-methyl-2-{4-[4-(trifluoromethoxy)benzyl]phenyl}quinolin-4(1H)-one (CK-2-68)	*Pf*NADH2 complex III (Q_o_ site)	(+)	(+)	(+)	[25,38,42,43,95]
7-Fluoro-3-methyl-2-{6-[4-(trifluoromethoxy)phenyl]pyridin-3-yl}quinolin-4(1H)-one (SL-2-64)	*Pf*NADH2 complex III	ND	(+)	ND	[38,42]
3-Methyl-2-{6-[4-(trifluoromethoxy)phenyl]pyridin-3-yl}quinolin-4(1H)-one (SL-2-25)	*Pf*NADH2 complex III	(+)	(+)	ND	[38,42,95]
5-Fluoro-3-methyl-2-[4-[[4-(trifluoromethoxy)phenyl]methyl]phenyl]-1*H*-quinolin-4-one (RYL-552)	*Pf*NADH2 complex III (Q_o_ site, Q_i_ site) *Pf*DHODH	ND	(+)	ND	[41,45,46]
5-Fluoro-3-methyl-2-[4-[[4-(trifluoromethoxy)phenyl]methyl]phenyl]-1-(N-ethyl-N-propylamino)-4(1H)-quinolone (RYL-581)	*Pf*NADH2 complex III (Q_o_ site, Q_i_ site)	ND	(+)	ND	[41]
1-Methyl-2-pentyl-4(1H)-quinolinone (MPQ)	*Pf*NADH2	ND	(−)	ND	[43]
RKA066	*Pf*NADH2 complex III (Q*_i_*site)	ND	(+)	ND	[47]
6-Chloro-7-methoxy-2-methyl-3-{4-[4-(trifluoromethyl) phenoxy]phenyl}quinolin-4(1H)-one (ELQ-300)	complex III (Q_i_ site)	(+)	(+)	(+)	[57,58,59,96]
[6-Chloro-7-methoxy-2-methyl-3-{4-[4-(trifluoromethyl) phenoxy]phenyl}quinolin-4-yl]oxymethyl ethyl carbonate (ELQ-331)	complex III (Q_i_ site)	(+)	(+)	(+)	[58,59,97]
6-Chloro-7-methoxy-2-methyl-3-[4-[4-(trifluoromethoxy)phenyl]phenyl]-1*H*-quinolin-4-one (ELQ-596)	complex III (Q_i_ site)	ND	(+)	ND	[61]
[6-Chloro-7-methoxy-2-methyl-3-[4-[4-(trifluoromethoxy)phenyl]phenyl]quinolin-4-yl]oxymethyl ethyl carbonate (ELQ-598)	complex III (Q_i_ site)	(+)	(+)	ND	[61]
WJM228	complex III (Q_o_ site)	(+)	(+)	(+)	[62]
ELQ-400	complex III (Q_o_ site, Q_i_ site)	(+)	(+)	(+)	[58,64,65,98]
**Acridine-related compounds**					
WR 249685	complex III	ND	(+)	ND	[57]
T111	complex III (Q_o_ site, Q_i_ site)	(+)	(+)	(+)	[66,67,99]
**Naphthoquinones**					
3-[4-(4-Chlorophenyl)cyclohexyl]-4-hydroxynaphthalene-1,2-dione (Atovaquone)	complex III (Q_o_ site)	(+)	(+)	(+)	[7]
1-(1,8-Dihydroxy-3-methylnaphthalen-2-yl) ethanone (Nepodin)	*Pf*NDH2	ND	(+)	ND	[48]
5-Hydroxy-2-methyl-1,4-naphthoquinone (Plumbagin)	complex II	ND	(+/−)	ND	[52,53,55]
**Pyridones**					
GW844520	complex III (Qi site)	(+)	(+)	ND	[42,47,69,100]
GSK932121	complex III (Qi site)	ND	(+)	ND	[42,47,57]
**Pyrimidine Azepines**					
PyAz90	complex III (Q_o_ site)	(+)	(+)	(+)	[70,101]
**Cyclopropyl carboxamides**					
MMV024397	complex III (Q_o_ site)	(+)	(+)	(+)	[56,71]
WJM280	complex III (Q_o_ site)	(+)	(+)	(+)	[71]
W466	complex III (Q_o_ site)	(+)	(+)	(+)	[71]
W499	complex III (Q_o_ site)	(+)	(+)	(+)	[71]
**Terpene compounds**					
Dobikin K	*Pf*NADH2 *Pf*DHODH	ND	(+)	ND	[49]
(4aS,6aS,11bR,13aS,13bS)-4,4,6a,9-Tetramethyl-1,2,3,4,4a,5,6,6a,11b,13b-decahydrobenzo[a]furo [2,3,4-mn]xanthen-11-ol (Siccanin)	complex II complex III	ND	(+)	ND	[51]
**Chalcones**					
(E)-3-[4-hydroxy-2-methoxy-5-(2-methylbut-3-en-2-yl)phenyl]-1-(4-hydroxyphenyl)prop-2-en-1-one (Licochalcone A)	complex II complex III	ND	(+)	ND	[54,55]
2-geranyl-2′,4′,3,4-tetrahydroxy-dihydrochalcone	*Pf*MQO	ND	(+)	ND	[88]
**Triazolopyrimidines**					
DSM265	*Pf*DHODH	(+)	(+)	(−)	[77,78,79,80,102,103,104,105]
DSM421	*Pf*DHODH	(+)	(+)	ND	[77]
**N-Alkyl-thiophene-2-carboxamides**					
Genz-667348	*Pf*DHODH	ND	(+)	ND	[42,79,82]
Genz-669178	*Pf*DHODH	ND	(+)	ND	[42]
**Pyrrole-based derivatives**					
DSM705	*Pf*DHODH	(+)	(+)	ND	[83,84]
DSM873	*Pf*DHODH	(+)	(+)	ND	[83,84]
**Pyrazole-based compounds**					
DSM1465	*Pf*DHODH	(+)	(+)	ND	[85]
**Other compounds**					
Ethyl-6-decyloxy-7-ethoxy-4-hydroxyquinoline-3-carboxylate (Decoquinate)	complex III (Q_o_ site)	(+)	(+)	(+)	[42,72]
Sodium azide (NaN_3_)	complex IV	ND	(+/−)	ND	[73]
Potassium cyanide (KCN)	complex IV	ND	(+/−)	ND	[73,74]
Almitrine	complex V	ND	(+)	ND	[55,75]
ITT-004	complex V	ND	(+)	ND	[76]
2,4,7-Trichloro-3,8-dihydroxy-1,9-dimethyl-6-(1-methyl-1-propen-1-yl)-11H-dibenzo[b,e][1,4]dioxepin-11-one (Nornidulin)	*Pf*MQO	ND	(+)	ND	[86]
4-Hydroxy-3-[(2E,6E)-3,7,11-trimethyldodeca-2,6,10-trienyl]chromen-2-one (ferulenol)	*Pf*MQO	ND	(+)	ND	[87]
Sodium fluoroacetate (NaFAc)	*Pf*ACO	ND	(−)	(+)	[17,89]
Mercaptosuccinic acid	*Pf*FH	ND	(+)	ND	[90]
ALA/SFC	oxidative stress	ND	(+)	ND	[92,93,94]
ART	membrane depolarization other unexplored mechanisms	(−)	(+)	(+)	[106,107]

## Data Availability

Not applicable.

## Abbreviations

The following abbreviations are used in this manuscript:

ABS	Asexual blood stages
ACTs	Artemisinin-based combination therapies
ADME	Absorption, distribution, metabolism, and excretion
ALA	5-Aminolevulinic acid
ANT	Adenine nucleotide translocator
ART	Artemisinin
ARTs	Artemisinin and its derivatives
CLogP	Calculated logarithm of the partition coefficient
CoQ	Coenzyme Q
CRISPR	Clustered Regularly Interspaced Short Palindromic Repeats
CTT1	Cytosolic catalase
CybL	Cytochrome b large
CybS	Cytochrome b small
Cyt *c*	Cytochrome c
DHA	Dihydroartemisinin
EC_50_	Half-maximal effective concentration
ELQs	Endochin-like quinolones
Fp	Flavoprotein
G3PDH	Glycerol 3-phosphate dehydrogenase
GSK	GlaxoSmithKline
HTS	High-throughput screening
IC_50_	Half-maximal inhibitory concentration
Ip	Iron–sulfur cluster protein
K_i_	Inhibitory constant
LSs	Liver stages
Mdh1	Mitochondrial malate dehydrogenase
mETC	Mitochondrial electron transport chain
MMV	Medicines for Malaria Venture
NaFAc	Sodium fluoroacetate
ND	Not detected
PD	Plasmodione
*Pf*ACO	*Plasmodium falciparum* aconitase
*Pf*BCKDH	*Plasmodium falciparum* branched chain ketoacid dehydrogenase complex
*Pf*CHA	*Plasmodium falciparum* Ca^2+^/H^+^ antiporter
*Pf*COCP	*Plasmodium falciparum* citrate/oxoglutarate carrier protein
*Pf*DHODH	*Plasmodium falciparum* dihydroorotate dehydrogenase
*Pf*ENT1	*Plasmodium falciparum* purine transporter
*Pf*FH	*Plasmodium falciparum* fumarate hydratase
*Pf*G3PDH	*Plasmodium falciparum* glycerol 3-phosphate dehydrogenase
*Pf*IDH	*Plasmodium falciparum* isocitrate dehydrogenase
*Pf*KDH	*Plasmodium falciparum* α-ketoglutarate dehydrogenase
*Pf*MQO	*Plasmodium falciparum* malate-quinone oxidoreductase
*Pf*NDH2	*Plasmodium falciparum* type II NADH dehydrogenase
*Pf*PiT	*Plasmodium falciparum* phosphate transporter
*Pf*SDH	*Plasmodium falciparum* succinate dehydrogenase
PHB	Prohibitin
Phyre2	Protein homology/analogY recognition engine
Pi	Inorganic phosphate
PK	Pharmacokinetic
PQ	Primaquine
RBCs	Red blood cells
ROS	Reactive oxygen species
SBS	Sexual blood stages
SCID	Severe combined immunodeficiency
SFC	Sodium ferrous citrate
SOD1	Cytosolic superoxide dismutase
SOD2	Mitochondrial superoxide dismutase
TCA	Tricarboxylic acid
TCPs	Target candidate profiles
WHO	World Health Organization
Δψ_m_	Mitochondrial membrane potential
